# Pyramidal tract neurons drive amplification of excitatory inputs to striatum through cholinergic interneurons

**DOI:** 10.1126/sciadv.abh4315

**Published:** 2022-02-09

**Authors:** Nicolás A. Morgenstern, Ana Filipa Isidro, Inbal Israely, Rui M. Costa

**Affiliations:** 1 Champalimaud Research, Champalimaud Centre for the Unknown, Lisbon 1400-038, Portugal.; 2 Departments of Pathology and Cell Biology, and Neuroscience, Taub Institute for Research on Alzheimer’s Disease and the Aging Brain, Columbia University, New York, NY 10027, USA.; 3 Departments of Neuroscience and Neurology, Zuckerman Mind Brain Behavior Institute, Columbia University, New York, NY 10027, USA.

## Abstract

Corticostriatal connectivity is central for many cognitive and motor processes, such as reinforcement or action initiation and invigoration. The cortical input to the striatum arises from two main cortical populations: intratelencephalic (IT) and pyramidal tract (PT) neurons. We report a previously unknown excitatory circuit, supported by a polysynaptic motif from PT neurons to cholinergic interneurons (ChIs) to glutamate-releasing axons, which runs in parallel to the canonical monosynaptic corticostriatal connection. This motif conveys a delayed second phase of excitation to striatal spiny projection neurons, through an acetylcholine-dependent glutamate release mechanism mediated by α4-containing nicotinic receptors, resulting in biphasic corticostriatal signals. These biphasic signals are a hallmark of PT, but not IT, corticostriatal inputs, due to a stronger relative input from PT neurons to ChIs. These results describe a previously unidentified circuit mechanism by which PT activity amplifies excitatory inputs to the striatum, with potential implications for behavior, plasticity, and learning.

## Teaser

Pyramidal tract neurons amplify striatal inputs via a polysynaptic circuit mediated by cholinergic interneurons.

## INTRODUCTION

In the brain, the connection from the cortex to the striatum is central for many cognitive and motor processes, such as learning new motor skills or selecting proper actions in response to internal or contextual changes (*1*–*3*). The striatum is the largest input nucleus to the basal ganglia, a group of interconnected subcortical nuclei that regulate brainstem, midbrain, and thalamocortical circuits, forming a long-range connectivity loop with the latter (*4*). In the striatum, about 95% of the neurons are γ-aminobutyric acid (GABA)–releasing (GABAergic) spiny projection neurons (SPNs) (*5*, *6*). Besides being the most abundant ones, SPNs are the only neuronal subtype projecting outside this structure (*5*), filtering the information that is outputted to downstream basal ganglia nuclei and, ultimately, modulating brainstem activity and thalamic and cortical feedback.

Synaptic input to striatum arises from most cortical areas and, to a lesser extent, from the thalamus, through highly organized excitatory long-range axons (*7*–*13*). Given that the striatum lacks intrinsic glutamatergic neurons (*14*), these inputs onto SPNs and other striatal neuronal subtypes are key for normal striatal function. Corticostriatal inputs are thought to convey motor and contextual signals to SPNs, information that is critical for proper action selection (*3*, *15*). Corticostriatal contacts are also the site for plasticity underlying striatal-dependent learning (*16*, *17*). Synaptic weight changes occur in excitatory contacts onto SPNs when mice learn a motor task (*18*, *19*). Moreover, movement disorders in humans and mouse models of diseases like Parkinson’s affect this connection (*20*–*23*), highlighting its importance.

The vast majority of the excitatory neurons projecting to the striatum are located in cortical layer 5 (L5) (*24*). However, they are a heterogeneous population and could be subdivided into two major categories: intratelencephalic (IT) and pyramidal tract (PT) neurons (*24*–*26*). Each subpopulation has characteristic intracortical laminar position and stereotyped axonal projection patterns, suggesting divergent functionality (*27*–*29*). The somata of IT neurons span cortical L5, and their axons extend within ipsi- and contralateral cortical areas and also to the ipsi- and contralateral striatum. On the other hand, PT somata are located in deep L5, and their corticofugal projections send collaterals to several ipsilateral subcortical structures, predominating those to the ipsilateral striatum (*30*). Moreover, while IT axons synapse onto PTs, direct PT contacts onto ITs are rare, suggesting a hierarchical IT➔PT anatomo-functional organization (*31*–*33*). These morphological and connectivity features, together with studies showing that IT and PT have distinct roles in action planning and execution (*29*), suggest that ITs are mostly involved in intracortical action preparation, while PTs trigger action execution by broadcasting a command signal throughout the multiple motor-related subcortical structures that they innervate.

The striatum is therefore the only noncortical structure where IT and PT pathways converge (*24*), synapsing onto both striatonigral and striatopallidal SPNs (*34*). Thus, understanding the differences between the signals that SPNs receive from these two key cortical afferents becomes crucial for tackling neuronal circuits supporting motor learning and behavior.

However, the direct cortex (Cx)➔SPN connection is not the sole determinant of striatal output. SPN spiking is tightly controlled by intrastriatal polysynaptic interactions with sparse local GABAergic and cholinergic interneurons (ChIs) (*35*). For instance, parvalbumin-expressing fast-spiking interneurons (FSIs), driven by cortical inputs, exert strong feed-forward inhibition onto SPNs, controlling their output (*36*–*39*). In turn, ChIs regulate striatal function by releasing acetylcholine that acts either through neuromodulatory muscarinic receptors onto SPNs (*40*) or through presynaptic nicotinic receptors regulating dopamine, GABA, and glutamate release (*41*–*46*). Recent studies showed nonuniform and highly specific organization of diverse cortical and thalamic inputs onto striatal interneurons, expanding our knowledge of their afferent connectivity (*10*, *13*, *35*, *47*). However, despite their critical influence on SPN spiking, it is still unknown whether striatal interneurons have biased inputs from IT versus PT neurons.

In this study, we used transgenic mouse lines, optogenetics, and slice electrophysiology to investigate the differences between IT and PT corticostriatal connectivity to the striatum. We found a previously unknown connectivity motif from PT neurons to ChIs to glutamate-releasing axons, running in parallel to the canonical monosynaptic Cx➔SPN connection. This motif conveys a delayed second phase of excitation to SPNs, through an acetylcholine-dependent glutamate release mechanism mediated by α4-containing nicotinic receptors, resulting in biphasic corticostriatal signals. Moreover, we found that these biphasic signals are a hallmark of PT, but not IT, corticostriatal inputs, due to their stronger relative input to ChIs. This work uncovers a previously unidentified circuit mechanism by which PT, but not IT neurons, amplify excitation to the striatum, with potential implications for behavior, plasticity, and learning.

## RESULTS

### PT corticostriatal inputs evoke biphasic responses onto SPNs

**Fig. 1.**
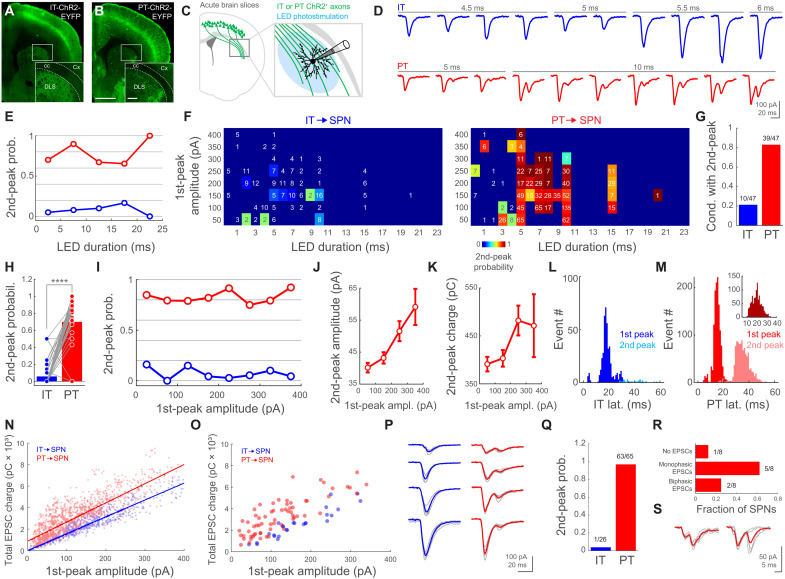
PT corticostriatal inputs evoke biphasic responses onto SPNs. (**A** and **B**) Images from IT- or PT-ChR2-EYFP mice. Scale bar, 1 mm; inset, 200 μm. (**C**) Experiment schematic. LED, light-emitting diode. (**D**) Examples of individual EPSCs upon IT/PT photostimulation. (**E**) Second-peak probability versus photostimulation duration. Biphasic/total trials: IT bins (blue), 13/260, 9/114, 2/20, 3/18, and 0/5 from 26 SPNs, 10 mice; PT bins (red), 206/294, 725/807, 86/128, 53/81, and 6/6 from 65 SPNs, 31 mice. (**F**) Matrices of biphasic EPSC probability. Trials/SPNs/mice: IT, 230/26/10; PT, 977/61/31. Numbers: trials per condition. (**G**) Proportion of conditions in (F) with at least one biphasic EPSC. (**H**) Paired biphasic EPSC probability for the 47 conditions in (F). *P* = 5.31 × 10^−8^; Wilcoxon signed-rank test. (**I**) Second-peak probability versus first-peak amplitude. Biphasic/total trials: IT bins (blue), 4/25, 0/38, 14/94, 4/93, 2/74, 3/56, 2/20, and 1/23 from 26 SPNs, 10 mice; PT bins (red), 213/251, 308/388, 211/267, 160/195, 107/117, 42/56, 23/29, and 12/13 from 65 SPNs, 31 mice. (**J** and **K**) Mean amplitude/charge of PT➔SPN second EPSC versus first-peak amplitude. (**L** and **M**) Histogram of first- and second-peak latencies. Trials/SPNs/mice: IT, 423/26/10. PT, 1316/65/31. Inset in (M): First- to second-peak latencies in individual trials; 1076/65/31. (**N** and **O**) Total EPSC charge versus first-peak amplitude. (N) Datapoints are trials. Trials/SPNs/mice: IT, 423/26/10. PT, 1316/65/31. Lines: linear fits. Slope with 95% confidence interval (CI): IT, 15.64 pC/pA (15.03 to 16.25); PT, 17.8 pC/pA (17.11 to 18.48). Intersect with 95% CI: IT, 20.18 pC (−105.3 to 145.6); PT, 888.8 pC (790.2 to 987.4). (O) Datapoints are SPNs. SPNs/mice: IT, 26/10. PT, 65/31. (**P**) Examples of single (gray) and mean (IT, blue; PT, red) EPSCs from individual SPNs. (**Q**) Biphasic EPSC probability for individual neurons. SPNs/mice: IT, 26/10. PT, 65/31. (**R**) SPN responses to M1➔DLS PT axonal photostimulation. SPNs/mice: 8/4. (**S**) Single (gray) and mean (red) traces from the SPNs that showed biphasic M1➔DLS EPSCs.

To investigate the differences between the signals that IT and PT corticostriatal neurons convey to SPNs, we crossed a transgenic mouse line expressing ChannelRhodopsin-2 (ChR2)–enhanced yellow fluorescent protein (EYFP) under the control of cre-recombianse [Ai32; *Rosa-CAG-LSL-ChR2(H134R)-EYFP-WPRE*] with a mouse line expressing cre-recombinase in either IT [Tlx3; *Tg(Tlx3-cre)PL58Gsat/Mmucd*] or PT [OE25; *Tg(Chrna2-cre)OE25Gsat/Mmucd*] cortical neurons. This resulted in the selective expression of ChR2-EYFP in one of these two neuronal populations (IT-ChR2-EYFP and PT-ChR2-EYFP, respectively), confirming their laminar location in cortical L5, as well as their long-range axonal projections into the dorsolateral striatum (DLS; Fig. 1, A and B). We then used whole-cell patch clamp to record excitatory postsynaptic currents (EPSCs) from SPNs in the DLS of acute brain coronal slices while wide-field photostimulating pathway-specific corticostriatal fibers (Fig. 1C). This is a well-established approach to study long-range connectivity in vitro, because ChR2-expressing axons remain photoexcitable despite losing branches or the connection to their parental soma during the slicing process (*48*–*53*). As expected, presynaptic photostimulation of identical power but different durations elicited postsynaptic responses of variable amplitude (Fig. 1D). Unexpectedly, IT and PT activation elicited responses with different characteristics. The stimulation of IT fibers evoked typical monophasic EPSCs, consistent with direct excitatory inputs from IT neurons. In turn, PT axon activation often elicited EPSCs with two distinguishable phases, suggesting an additional excitatory component (Fig. 1D).

To quantify the probability of evoking a second peak in IT and PT➔SPN EPSCs, we used a threshold to detect the second phase of corticostriatal signals (Materials and Methods). We found that, throughout a wide range of photostimulation conditions, the probability of evoking biphasic EPSCs was higher for PT than for IT inputs (Fig. 1E). These experiments also evidenced that, although the photostimulation duration and the amplitude of the first peak of the EPSC positively correlate (mean correlation coefficient, *r* = 0.48 ± 0.05; *n* = 73 SPNs), IT➔SPN EPSCs progress with a slope ~5 times steeper than PT➔SPN EPSCs (IT, 154.83 ± 26.95 pA/ms, *n* = 24 SPNs; PT, 30.36 ± 11 pA/ms, *n* = 49 SPNs; *P* = 4.04 × 10^−7^, Wilcoxon rank sum test, *z* = 5.07). As a consequence, PT fibers required, on average, longer illuminations than IT axons for evoking EPSCs of similar amplitude (IT, 6.05 ± 0.28 ms; range, 0.1 to 30; and *n* = 423; PT, 8.52 ± 0.11 ms; range, 0.3 to 22; and *n* = 1316; *P* = 1.13 × 10^−44^, Wilcoxon rank sum test, *z* = −14.02). It is important to note that biphasic responses were not the result of long illuminations, because full-field photostimulation through the objective lens with light pulses of 1 to 2 ms elicited similar EPSCs when photostimulating PT axons (fig. S1).

One possible explanation for the different photostimulation/amplitude relationship in IT and PT➔SPN EPSCs above-mentioned is a dissimilar level of ChR2 expression between the transgenic lines. Therefore, to assess the probability of evoking biphasic responses under more comparable conditions, we next restricted this analysis to IT and PT➔SPN EPSCs of equivalent amplitude elicited by similar illuminations (Fig. 1F). We detected 47 conditions where EPSC first-peak amplitudes and light durations were similar for IT and PT stimulations. Notably, biphasic responses were more frequent upon PT than IT activation (Fig. 1G). Moreover, when comparing individual conditions, we found that the probability of evoking a second peak was significantly higher for PT than for IT➔SPN EPSCs (Fig. 1H). Together, these results confirm that, despite differential responsiveness to light, biphasic responses are more likely evoked by PT than by IT corticostriatal inputs.

Next, we studied how the first and second phases of the EPSCs relate to each other. For this purpose, we sorted the individual IT and PT➔EPSC trials by the amplitude of their first peak. The probability of evoking a second phase on SPN EPSCs was consistently higher for PT than for IT stimulation across the whole range of first-peak amplitudes explored (Fig. 1I). In addition, we found that both the amplitude and the charge of the second phase increased with the increase in the amplitude of the first peak (Fig. 1, J and K). Then, we measured the latencies from the start of illumination to the first and second peaks of the EPSCs (Fig. 1, L and M). We found that the overall latency to the first peak was shorter for PT➔SPNs when compared to IT➔SPNs (PT, 15.32 ± 0.11 ms and *n* = 1316 trials; IT, 18.39 ± 0.24 ms and *n* = 423 trials; *P* = 1.46 × 10^−60^, Wilcoxon rank sum test, *z* = 16.42). Moreover, this analysis showed a mean delay of 19.86 ± 0.14 ms (*n* = 1076 trials) from the first to the second peak evoked by PT photostimulation (Fig. 1M, inset). Last, we wondered how the identity of the presynaptic inputs affects the total charge that the postsynaptic SPNs receive. One possibility is that, because of a higher probability for evoking biphasic responses, PT➔SPN EPSCs transfer more charge to their postsynaptic targets than IT➔SPN EPSCs of similar amplitude. Alternatively, similar responses could transfer the same amount of charge but with a different temporal profile. We found that EPSCs with similar first-peak amplitude transfer more charge from PT than from IT inputs (Fig. 1N). Together, these results suggest that PT➔SPN inputs, by reliably conveying an additional, delayed, and proportional second EPSC phase, are more efficient than IT➔SPN inputs for exciting SPNs.

We next investigated how the findings from this dataset, displayed above in a trial-by-trial basis, were reflected at the level of individual neurons. Consistently, we found that, when comparing EPSCs in a similar range of first-peak amplitudes, individual SPNs receive more charge from PT than from IT presynaptic neurons (Fig. 1O). For this analysis, we first averaged five consecutive photostimulation trials, resulting in a mean EPSC for each SPN (Fig. 1P). This approach also allowed us to calculate the neuron-based EPSC second-peak response probability. In line with the results above, we found a very low probability for evoking biphasic responses when activating IT fibers but a highly reliable occurrence of EPSC second phases when stimulating PT inputs (Fig. 1Q).

PT-cre OE25 mouse line targets cre-recombinase expression to PT neurons from most cortical areas (*54*). However, its specificity is not totally restricted to these neurons, showing cre-expression in corticothalamic neurons and, to a lesser extent, in some neurons outside the cortex (*54*)(www.gensat.org). We dismissed any contribution of corticothalamic activation to our findings because in striatal slices, thalamic neurons are absent; thus, polysynaptic effects mediated by the cortex➔thalamus➔striatum circuit are not likely. We therefore designed an experiment to rule out the possibility that the EPSC second phase is elicited by ChR2-expressing long-range axons not originated in the cortex. For this purpose, we injected a cre-dependent adeno-associated virus (AAV) in the motor cortex (M1) of OE25 mice, limiting the expression of ChR2 to PT neurons in this area and, ~8 weeks later, we recorded from SPNs in the DLS. We failed to evoke biphasic EPSC in most of the SPNs (Fig. 1R), which was not totally unexpected because the AAV injection restricts the expression of ChR2 to a small subset of the neuronal population labeled in the PT-ChR2-EYFP mice, resulting in sparser ChR2^+^ axons in the striatum. However, in 25% of the SPNs, we evoked biphasic EPSCs (Fig. 1, R and S), proving that exclusive photostimulation of PT corticostriatal axons is sufficient to mimic the responses found in the double transgenic line. In summary, we found that IT and PT corticostriatal signals are different, with IT inputs evoking mostly monophasic responses and PT inputs reliably eliciting two sequential excitatory signals that result in biphasic EPSCs onto SPNs.

### Corticostriatal PT➔SPN EPSC second peak is mediated by striatal ChIs

In light of our findings, we hypothesized that, whereas the first IT and PT EPSC peak is mediated by direct cortical input to SPNs, the second phase reflects intrastriatal polysynaptic excitation, preferentially elicited by PT long-range axons. We dismissed a potential contribution of intracortical polysynaptic interactions, because most cortical axons innervating the DLS are severed from their somas upon coronal slice preparation, due to the anatomy of cortical projections. In addition, focal presynaptic phostostimulation with a laser beam in the near vicinity of the recorded SPNs reliably evoked biphasic PT➔SPN EPSCs (fig. S2A), further supporting a role for local intrastriatal interactions in the late phase of the EPSC.

**Fig. 2.**
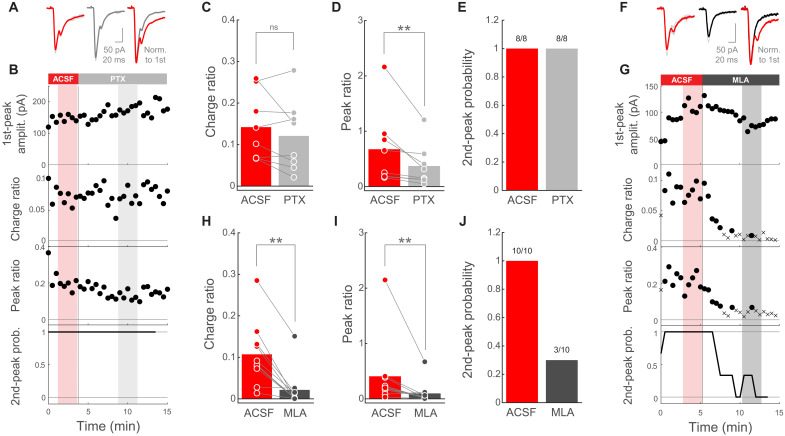
Corticostriatal PT➔SPN EPSC second peak is mediated by striatal ChIs. (**A**) Left: EPSCs from a representative SPN when photostimulating PT fibers in artificial cerebrospinal fluid (ACSF) (red) or PTX (gray). Right: Normalized mean traces. (**B**) Amplitude of the EPSC first-peak, charge ratio, peak ratio, and second-peak probability as a function of time, for the whole experiment in (A). Probability was calculated within a moving window of three consecutive trials. Shadowed areas highlight the individual trials averaged for each condition in (A). (**C** and **D**) Charge ratio and peak ratio for individual SPNs in ACSF and PTX. SPNs/mice: 8/5. *P* = 0.3125 (C); *P* = 0.0078125 (D); Wilcoxon signed-rank test. (**E**) Second-peak response probability for individual SPNs before and after PTX. SPNs/mice: 8/5. (**F**) Left: EPSCs from a representative SPN when photostimulating PT fibers in ACSF (red) or MLA (black). Right: Normalized mean traces. (**G**) EPSC metrics as a function of time for the whole experiment in (F). For charge and peak ratio: circles, EPSC with second peak; crosses, EPSC without second peak. Probability calculated as in (B). Shadowed areas highlight the trials averaged in (F). (**H** and **I**) Charge ratio and peak ratio for individual SPNs in ACSF and MLA. SPNs/mice: 10/7. *P* = 0.0019531 (H); *P* = 0.0058594 (I); Wilcoxon signed-rank test. (**J**) Second-peak response probability for individual SPNs before and after MLA. SPNs/mice: 10/7.

More than 95% of striatal neurons are GABAergic SPNs (*5*, *6*). Moreover, with exception of a small population of acetylcholine-releasing ChIs, all other local interneurons also release GABA (*35*). Because in our experimental conditions SPNs were clamped at a membrane potential below the chloride reversal potential (SPN *V*_holding_ = −80 mV and *E*_cl_ = −75.6 mV), GABA receptor activation would result in depolarizing rather than hyperpolarizing currents (*55*). For this reason, we first investigated whether GABAergic neurotransmission is underlying the second phase of PT➔SPN excitation. For this purpose, we photostimulated PT axons while monitoring SPN EPSCs in the absence or presence of the GABA_A_ receptor antagonist picrotoxin (PTX; 100 μM). Because the charge and amplitude of the EPSC second-phase increase with the amplitude of the first peak (Fig. 1, J and K), we calculated the charge ratio and the peak ratio to relate the magnitude of both phases (Materials and Methods). These normalized metrics, as well as the second-peak response probability, resulted robust to fluctuations in the amplitude of the first peak (fig. S3), allowing us to make comparisons across trials within the same SPN, as well as across different SPNs. Notably, the addition of PTX to the extracellular solution had no effect on the charge ratio (Fig. 2, A to C). Although PTX elicited a moderate decrease in the peak ratio (Fig. 2D), the magnitude of such decrease was not enough to fully suppress the second phase of EPSCs in any of the recorded SPNs (Fig. 2, B and E). This mild change in peak ratio might be reflecting inhibitory/disinhibitory circuit interactions as a consequence of altering intrastriatal GABA signaling, the major neurotransmitter in this structure. Together, these data suggest little involvement of GABAergic transmission in the second phase of PT➔SPN responses. In addition, the PT➔SPN second phase did not change its polarity when the postsynaptic membrane was clamped above the chloride reversal potential (fig. S2B), further supporting that it is not mediated by GABA.

Having ruled out the role of GABAergic interneurons, we postulated that ChIs could mediate this local excitation triggered by PT. To test this, we recorded SPNs while photostimulating PT axons without or with the broad nicotinic acetylcholine receptor antagonist methyllycaconitine (MLA; 1 μM) in the bath (*56*). MLA significantly reduced the charge ratio, the peak ratio, and the second-peak response probability of PT➔SPN responses (Fig. 2, F to I), which partially recovered after MLA wash out (fig. S3, B and C). Notably, MLA preferentially affected the second, rather than the first, phase of these EPSCs (figs. S3 and S4), indicating that it did not reduce overall excitability and that the EPSC first and second phases are regulated by different mechanisms. Moreover, when comparing the magnitude of the reduction exerted by MLA or PTX, we found a significantly stronger effect of the cholinergic blocker on both the charge ratio and the peak ratio (fig. S5, A and B). Furthermore, MLA reduced the PT➔SPN EPSC second phase to undetectable levels in most of the recorded neurons (Fig. 2J), implicating ChIs in these delayed signals. In similar experiments using the muscarinic general blocker atropine (10 μM), the EPSC second phase was not affected (fig. S5, C to F), showing that nicotinic but not muscarinic receptors are at the core of this excitatory mechanism. In summary, our data strongly suggest that the second excitatory component of the biphasic PT➔SPN responses is mediated by local striatal ChIs acting through nicotinic receptors.

### ChIs indirectly excite SPNs via an acetylcholine-dependent glutamate release mechanism

We next wanted to address the location of the nicotinic receptors mediating the second phase of PT➔SPN EPSCs. One possibility is that ChIs form direct monosynaptic contacts onto SPNs supporting nicotinic transmission, whose existence, to our understanding, is not described in the literature (*35*, *57*). Alternatively, ChIs could indirectly excite SPNs by activating nicotinic receptors onto presynaptic glutamate-releasing axons (*45*, *46*, *53*, *58*–*60*). To distinguish these two possibilities, we used a double transgenic mouse line [*Choline-Acetyl Transferase (ChAT)-Cre x Ai32*] where ChR2 is expressed in acetylcholine-releasing neurons (ChAT-ChR2-EYFP). We verified the specific expression of ChR2-EYFP in striatal ChIs using immunofluorescence and confocal microscopy (Materials and Methods). In all tested subjects, we found that almost all cases of striatal neurons expressing ChR2-EYFP were ChAT^+^, with a proportion of ChR2-EYFP^+^ neurons coexpressing ChAT higher than 0.9 in six of seven mice. These data confirmed that, in the ChAT-ChR2-EYFP line, ChR2 expression in the striatum is highly restricted to ChIs. Hence, because the cholinergic innervation of the striatum is dominated by local interneurons rather than by extrinsic innervation (*40*, *41*, *61*, *62*), we concluded that photostimulation in this preparation would reveal the impact of the activation of striatal ChIs.

**Fig. 3.**
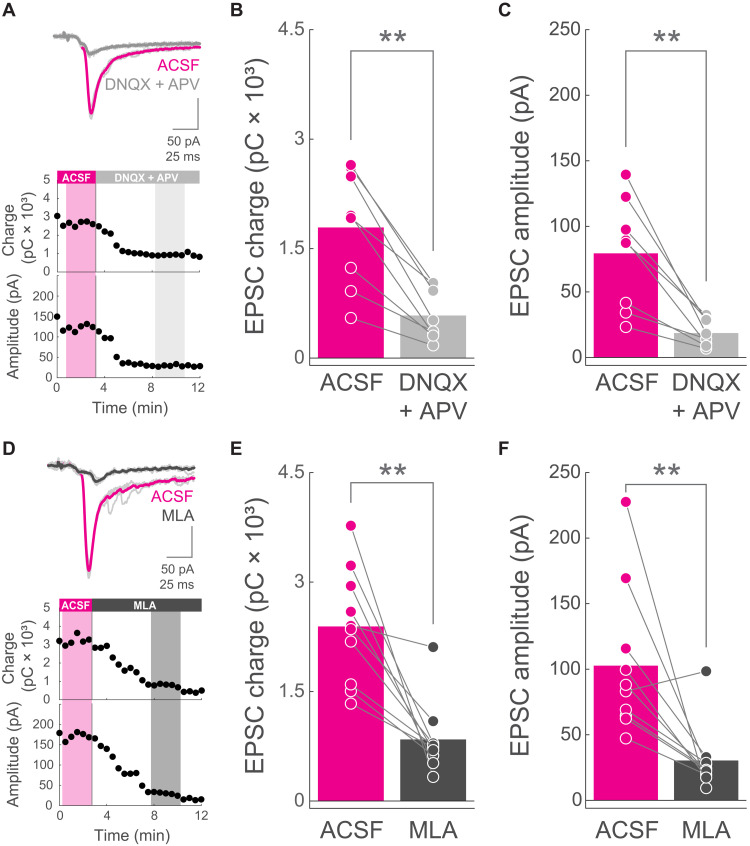
ChIs indirectly excite SPNs via an acetylcholine-dependent glutamate release mechanism. (**A**) Top: Example of an individual experiment showing EPSCs from an SPN when photostimulating ChAT-ChR2-EYFP neurons before (ACSF, magenta) and after DNQX and APV (gray). Bottom: EPSC charge and peak amplitude as a function of time, for the same experiment of the traces above. Shadowed areas highlight the individual trials averaged for each condition (magenta, ACSF; light gray, DNQX and APV). (**B** and **C**) EPSC charge (B) and amplitude (C) for individual SPNs in ACSF (magenta) and DNQX and APV (gray) conditions. *n* = 8 SPNs from four ChAT-ChR2-EYFP mice. Bars represent means. *P* = 0.0078125 (B); *P* = 0.0078125 (C); Wilcoxon signed-rank test. (**D**) Top: Example of an individual experiment showing EPSCs from an SPN when photostimulating ChAT-ChR2-EYFP neurons before (ACSF, magenta) and after MLA (black). Bottom: EPSC charge and peak amplitude as a function of time, for the same experiment of the traces above. Shadowed areas highlight the individual trials (thin light-gray traces above) averaged for each condition (magenta, ACSF; dark gray, MLA). (**E** and **F**) EPSC charge (E) and amplitude (F) for individual SPNs in ACSF (magenta) and MLA (black) conditions. *n* = 10 SPNs from five ChAT-ChR2-EYFP mice. Bars represent means. *P* = 0.0019531 (E); *P* = 0.0039063 (F); Wilcoxon signed-rank test.

We could reliably evoke EPSCs on the recorded SPNs when photoactivating ChIs (Fig. 3, A and D). In line with previous studies (*60*), ChI➔SPN EPSCs exhibited long latencies to peak (31.48 ± 1.6, *n* = 20 SPNs from nine mice) and were preceded in ~10 ms (9.84 ± 0.94 ms) by a small inward current (mean amplitude, 23.46 ± 3.97) in 16 of 20 SPNs (Fig. 3, A and D, and fig. S6, A and D). These features are compatible with an indirect, probably polysynaptic, mechanism underlying the main ChI➔SPN EPSC component. If this is the case, and ChI➔SPN EPSCs do require acetylcholine-induced release of glutamate from presynaptic terminals, then these responses should be modulated by glutamate receptor blockers. Alternatively, if ChIs directly contact postsynaptic nicotinic receptors onto SPNs, then ESPCs should be insensitive to the glutamatergic receptor antagonists. We found that the addition of the AMPA and *N*-methyl-d-aspartate glutamate receptor blockers 6,7-dinitroquinoxaline-2,3-dione (DNQX; 10 μM) and (2*R*)-amino-5-phosphonovaleric acid (APV; 50 μM), respectively, significantly reduced the charge and amplitude of ChI➔SPN EPSCs (Fig. 3, A to C). These results show the necessity of glutamatergic transmission for ChI➔SPN EPSCs and support the activation of nicotinic receptors onto presynaptic glutamate-releasing afferents as the mechanism underlying ChI➔SPN excitation.

However, in the DLS, ChIs corelease glutamate together with acetylcholine (*63*). Thus, monosynaptic ChI➔SPN glutamatergic transmission, which should be resistant to nicotinic receptor blockade, could directly mediate these EPSCs. To test this, we stimulated ChIs before and after the addition of MLA to the bath. We found that both the charge and the amplitude of ChI➔SPN EPSCs were significantly reduced by MLA (Fig. 3, D to F), suggesting an excitatory mechanism that resembles the one involved in PT➔SPN EPSC second phases (Fig. 2). While these experiments do not fully discard some direct ChI➔SPN glutamate contribution, they do highlight the necessity of nicotinic receptor activation to mediate most of the excitation conveyed from ChIs to SPNs, further supporting a scenario where acetylcholine acts on presynaptic terminals from glutamate-releasing fibers.

The small inward current preceding the EPSC peak became more evident after the addition of glutamatergic or nicotinic receptor antagonists, because although its amplitude was significantly modulated, its area was resistant to these blockers (fig. S6). Therefore, these findings indicate that the neurotransmitters and/or the receptors involved in this early EPSC phase are, at least in part, different from the ones mediating the main EPSC component characterized here. Together, these results show that ChIs mediate the PT➔SPN EPSC second peak through acetylcholine-dependent glutamate release from long-range axons innervating DLS.

### PT➔SPN EPSC second peak requires α4-containing nicotinic receptor activation

**Fig. 4.**
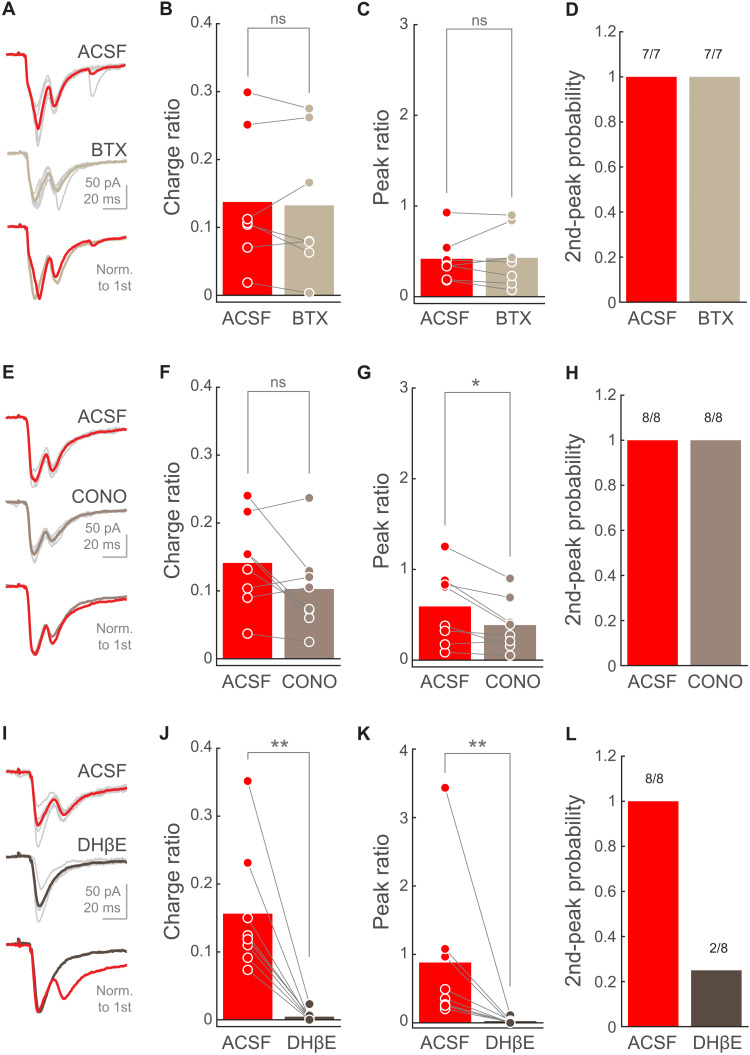
PT➔SPN EPSC second peak requires α4-containing nicotinic receptor activation. (**A**, **E**, and **I**) EPSCs from a representative SPN when photostimulating PT fibers in ACSF (top) or nicotinic blocker condition (middle). Thin light-gray traces are the five individual trials corresponding to the thicker mean traces. Bottom: Mean traces normalized to the first peak. (**B**, **F**, and **J**) Charge ratio for individual SPNs in ACSF or nicotinic blocker condition. BTX, *n* = 7 SPNs from three mice; CONO, *n* = 8 SPNs from three mice; DHβE, *n* = 8 SPNs from four mice. Bars represent means. *P* = 0.57813 (B); *P* = 0.25 (F); *P* = 0.0078125 (J); Wilcoxon signed-rank test. (**C**, **G**, and **K**) Peak ratio for individual SPNs in ACSF or nicotinic blocker condition. BTX, *n* = 7 SPNs from three mice; CONO, *n* = 8 SPNs from three mice; DHβE, *n* = 8 SPNs from four mice. Bars represent means. *P* = 0.6875 (C); *P* = 0.023438 (G); *P* = 0.0078125 (K); Wilcoxon signed-rank test. (**D**, **H**, and **L**) Second-peak response probability upon photostimulation of PT fibers for individual SPNs before and after the addition of the selective nicotinic antagonist. BTX, *n* = 7 SPNs from three mice; CONO, *n* = 8 SPNs from three mice; DHβE, *n* = 8 SPNs from four mice.

We next wanted to characterize the precise ligand binding subunit (α-subunit) present in the nicotinic receptors supporting the PT➔SPN EPSC second phase. For this purpose, we recorded SPNs and photostimulated PT axons in the absence and presence of selective α-subunit antagonists to assess their impact on the EPSC second peak, as in Fig. 2. We found that, when blocking α7-containing receptors with α-bungarotoxin (BTX; 100 nM) or α6-containing receptors with α-conotoxin PIA (CONO; 10 nM), the EPSC second phase remained mostly unaffected (Fig. 4, A to H). In notable opposition, when α4 subunit was selectively antagonized with dihydro-β-erythroidine (DHβE; 1 μM) the EPSC charge and peak ratio were significantly reduced (Fig. 4, I to K), leading to a complete elimination of the second peak in three-quarters of the recorded SPNs (Fig. 4L). Together, these experiments show that the delayed excitation evoked by PT stimulation is strongly dependent on the selective activation of α4-containing nicotinic receptors on presynaptic glutamate-releasing axons reaching the DLS.

### PT neurons provide stronger relative inputs to ChIs than IT neurons

Next, we investigated the connectivity rules operating for each corticostriatal pathway. We wondered whether differences in the relative input strength to ChIs and SPNs could explain the biphasic excitation evoked by PT but not by IT activation. Because cortical input stimulation recruits ChIs, increasing the intrastriatal levels of acetylcholine (*47*, *64*, *65*), we hypothesized that PT➔ChI connection is relatively stronger than IT➔ChI connection. In this manner, provided similar input to SPNs, PT inputs would more reliably trigger ChI➔SPN excitation, favoring biphasic corticostriatal responses. Thus, we designed an experiment to test whether cortical IT and PT neurons monosynaptically contact striatal ChIs, and if so, how the connection strength from each pathway is distributed between ChIs and SPNs.

**Fig. 5.**
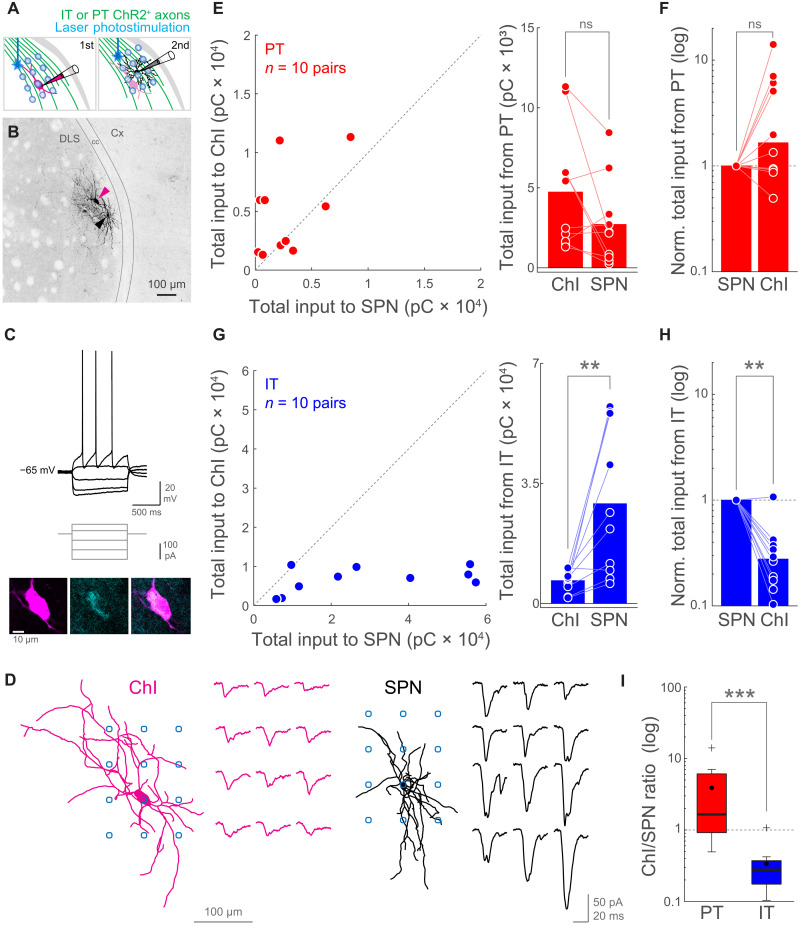
PT neurons provide stronger relative inputs to ChIs than IT neurons. (**A**) Experiment schematic. First, a ChI was recorded while IT- or PT-ChR2^+^ presynapses were photostimulated following a 3 × 4 grid pattern using a one-photon blue laser in TTX and 4-AP. Second, a neighboring SPN was recorded while photostimulating with the same pattern and identical illumination conditions. (**B**) Image showing a pair of biocytin-filled ChI (magenta arrowhead) and SPN (black arrowhead) from an IT-ChR2-EYFP mouse. (**C**) Top: Input-output curve of the ChI in (B). Middle: Current injection steps. Bottom: Confocal plane showing the soma of that ChI (left, magenta), its immunoreactivity to anti-ChAT antibodies (center, cyan), and both signals overlapped (right). (**D**) Reconstructions of the neurons in (B) with the relative position of their photostimulation grid (blue circles) and their correspondent matrix of IT➔ChI or IT➔SPN EPSCs. (**E** and **G**) Total input charge transferred to ChI-SPN pairs. Datapoints: left, pairs; right, neurons. PT: *P* = 0.16016; pairs/mice: 10/9; IT: *P* = 0.0039063; pairs/mice: 10/10. Wilcoxon signed-rank test. (**F** and **H**) Normalized total input to ChI-SPN pairs. Bars represent medians. PT➔SPN versus PT➔ChI, *P* = 0.10547; pairs/mice: 10/9. IT➔SPN versus IT➔ChI, *P* = 0.0039063; pairs/mice: 10/10. Wilcoxon signed-rank test. (**I**) Box plot comparing the normalized input to ChIs (ChI/SPN ratio) from PT and IT. *P* = 0.00058284; Wilcoxon rank sum test, *z* = 3.44. Pairs/mice: PT, 10/9; IT, 10/10. Horizontal line, median; black circle, mean; box edges, 25th/75th percentile; whiskers, maximum and minimum excluding outliers; crosses, outliers.

In brains slices of IT- or PT-ChR2-EYFP mice, we first patched a ChI in the DLS (Fig. 5, A and B). We initially recorded visually identified putative ChIs and confirmed their cholinergic identity by assessing their typical electrophysiological properties (depolarized resting membrane potential, presence of sag upon hyperpolarization, spike half-width > 1 ms, and regular spiking; Fig. 5C and fig. S7) in drug-free extracellular solution. We further confirmed the cholinergic phenotype of the recorded neurons by their characteristic larger somatic area (ChIs, 239.86 ± 11.79 μm^2^; range, 146.32 to 356.6; and *n* = 19; SPNs, 108.46 ± 6.53 μm^2^; range, 59.57 to 160.62; and *n* = 20; *P* = 1.6 × 10^−7^, Wilcoxon rank sum test, *z* = −5.24; Fig. 5B) and their reactivity to ChAT immunolabeling (16 of 19 ChIs were recovered and identified as ChAT^+^; Fig. 5C).

After that, we added 4-aminopiridine (4-AP; 100 μM) and tetrodotoxin (TTX; 1 μM) to the extracellular solution. This drug combination suppresses spiking while preserving ChR2^+^ synaptic terminal ability to release neurotransmitter when photostimulated, thus restricting EPSCs only to monosynaptic contacts (*49*–*52*). Under these conditions, we photostimulated IT or PT corticostriatal presynapses with a blue laser following a grid pattern over the dendrites of the postsynaptic ChI (Fig. 5D). For each neuron, we computed the total input charge by summing all EPSCs. Because both IT and PT corticostriatal pathway stimulation elicited monosynaptic responses on ChIs, we calibrated the illumination conditions to evoke comparable IT➔ChI and PT➔ChI total input charge across experiments (IT, 6.77 ± 1.01 pC × 10^3^; range, 1.69 to 10.57; and *n* = 10; PT, 4.76 ± 1.34 pC × 10^3^; range, 1.31 to 11.32; and *n* = 9; *P* = 0.2775, Wilcoxon rank sum test). Ideally, we would have recorded first from SPNs and calibrated photostimulation to evoke similar IT➔SPN and PT➔SPN input. However, the need for confirming ChIs electrophysiological phenotype in the absence of drugs forced us to record first from ChIs. Right after assessing the total input to the ChI, we recorded a neighboring SPN (less than 100 μm apart; Fig. 5, A and B, and fig. S7E) and repeated the photostimulation protocol with identical illumination conditions (Fig. 5D). Because neighboring neurons could potentially sample from the same population of afferent axons, with this experimental design we could compare the pathway-specific input strength balance to ChI-SPN pairs, irrespective of ChR2 expression level variations across slices (*49*–*52*).

We found that the input strength is differentially weighted for each corticostriatal pathway. While PT inputs exhibited similar total strengths across ChIs and SPNs (Fig. 5, E and F), the IT connection was significantly biased toward the SPNs (Fig. 5, G and H). To further quantify how the connection strength of each pathway distributes between ChIs and SPNs, we normalized the corticostriatal total input that a given ChI-SPN pair receives to the total input of that SPN (Fig. 5, F and H). We found that the PT➔ChI relative input strength is variable, with a population median of 1.66 times the PT➔SPN input (Fig. 5F). Contrarily, IT➔ChI relative input strength was consistently weaker, with a population median of 0.28 times the IT➔SPN input (Fig. 5H). These data strongly suggest that IT and PT pathways follow different connectivity rules when synapsing onto striatal neurons. Moreover, the normalized PT➔ChI input was one order of magnitude stronger than the normalized IT➔ChI input (Fig. 5I), supporting a model where, provided similar input to SPNs, PT neurons are more likely to recruit ChIs than IT neurons. These differences in IT and PT connectivity could not be explained by differences in the size of the dendrites of the recorded ChIs (fig. S7G) nor by the distance between ChIs and SPNs within the same pair (fig. S7E).

In addition, these experiments allowed us to compare IT➔SPN and PT➔SPN monosynaptic responses. We found that 4-AP and TTX abolished the differences between IT and PT afferents in their ability to evoke biphasic EPSCs and in the amount of charge that they transfer to SPNs (fig. S8). This evidence further supports that the EPSC second phase is not mediated by direct corticostriatal synaptic contacts. Therefore, these data provide independent evidence that PT➔SPN EPSC second peak requires intrastriatal polysynaptic interactions to occur.

**Fig. 6.**
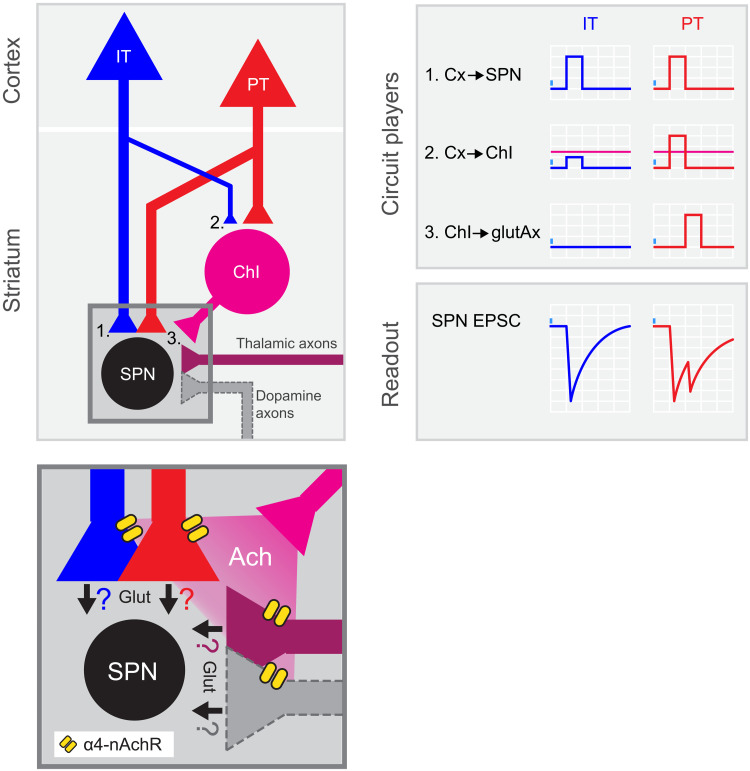
PT neurons amplify excitatory inputs to striatum through ChIs. Summary of the results: left, circuit diagram proposed for corticostriatal connectivity. IT and PT cortical neurons project to both SPNs (1) and ChIs (2). While PT➔SPN (1, red) and PT➔ChI (2, red) input strength is similar, IT➔ChI (2, blue) connection is weaker than IT➔SPN (1, blue). Within the striatum, ChIs convey excitation to SPNs by recruiting long-range glutamate-releasing terminals reaching DLS through α4-containing nicotinic receptors (3 and inset). Inset shows the putative axonal sources of glutamate release during the second EPSC peak. Ach, acetylcholine; α4-nAchR, α4-containing nicotinic receptors; Glut, glutamate. Right: Schematic of the activation of the different circuit players upon IT or PT photostimulation and its impact on the recorded SPNs. Magenta horizontal lines represent ChI spiking threshold.

In conclusion, our findings support the coexistence of parallel IT and PT excitatory corticostriatal circuits. On one hand, there is the canonical direct long-range connection from cortical neurons to SPNs, which photoactivation elicits the monosynaptic first phase of the IT➔SPN and PT➔SPN EPSCs (Fig. 6, left). On the other hand, we dissected the direct long-range projection to striatal ChIs into IT and PT pathways, showing a higher relative input strength for PT➔ChIs than for IT➔ChIs (Fig. 6, left). As a consequence, we propose that, given photostimulations that evoke similar EPSC first-peak amplitudes onto SPNs, PT inputs are more likely to recruit ChI➔SPN excitation (Fig. 6, right). Together with our previous data, these results support a corticostriatal Cx➔ChIs➔ glutamate-releasing axons (glutAx)➔SPNs excitatory motif strongly biased toward PT and explain why these inputs are more efficient than IT inputs to evoke biphasic excitation onto SPNs.

## DISCUSSION

Our data uncover a previously unknown circuit mechanism by which IT and PT corticostriatal inputs differentially impact SPNs (Fig. 6). We found a corticostriatal excitatory circuit, predominantly supported by the PT➔ChIs➔glutAx➔SPNs motif, running in parallel to the canonical excitatory monosynaptic connection from the cortex to SPNs. The photoactivation of that motif evokes a second phase of glutamate release onto SPNs, mediated by acetylcholine-induced activation of α4-containing nicotinic receptors at presynaptic terminals in the DLS, and resulting in biphasic corticostriatal signals (Fig. 6). These signals are preferentially evoked by PT, rather than by IT activation, because of a stronger PT➔ChI relative input strength that is more likely to recruit ChI➔SPN excitation (Fig. 6). In summary, this study dissects the IT and PT corticostriatal connectivity to ChIs, unraveling a circuit motif originating in PT command signals, which boosts SPN excitation. Therefore, the motif unraveled here opens new vistas into how PT versus IT corticostriatal signals can affect movement, plasticity, and learning.

The corticostriatal connection has been extensively studied (*7*–*13*). However, this connection was usually assumed homogeneous, neglecting the differences between the diversity of cortical inputs impinging onto striatal SPNs and interneurons. In that sense, previous functional studies using electrical or optogenetic stimulation of corticostriatal fibers and testing its impact onto SPNs would have predominantly recruited the denser bilateral IT pathway, occluding the details of the sparser PT➔SPN connection described here. We overcame that limitation by using mouse lines allowing population-specific control of axonal spiking (*54*). We found that, in parallel to the direct IT/PT➔SPN connection (*34*), both IT and PT pathways contact ChIs with different relative strength, indicating target selectivity of corticostriatal pathways. A recent study testing ipsi- and contralateral striatal innervation from motor and somatosensory cortices showed that ChIs are exclusively contacted by ipsilateral axons, most likely reflecting predominant PT inputs. In that same study, FSIs, the canonical striatal feed-forward inhibitory interneurons (*37*, *38*) were proven to be highly innervated by bilateral corticostriatal fibers, indicating strong IT connectivity (*13*). Such observation is suggestive of, at least, some degree of corticostriatal IT➔FSI and PT➔ChI specificity, which is partially demonstrated here by our result showing stronger PT relative input strength to ChIs (Fig. 5I). Although further studies are necessary, this idea becomes especially relevant in the context of a model where ITs are preparatory and PTs broadcast the action command to many motor-related structures (*29*). It is, then, tempting to speculate that ITs may permit action preparation by triggering up-states onto action-specific SPNs while silencing action-unrelated SPNs through FSIs. Subsequent PT signals might be key for transitioning from up-states to spikes in the action-related SPNs commanding execution. In this scenario, the amplification of the excitation exerted by PT➔ChIs➔glutAx➔SPNs, by boosting postsynaptic membrane depolarization and input integration, could help securing SPN spiking and downstream information flow. A possible reason why this second excitatory phase was not detected in the scarce functional studies that have tested PT➔SPN connectivity to date may be on cortical neurons heterogeneity. Recent studies showed that both IT and PT populations could be genetically subdivided into several subclasses with different synaptic targets (*66*, *67*). Thus, because in those cases focal subcortical injections of retrogradely labeling virus were used to achieve PT expression of ChR2 (*34*), it is likely that, in previous studies, only a subset of the neurons labeled in the PT-ChR2-EYFP mice were recruited by photostimulation. A similar scenario may underlie the variable EPSCs that we evoked when driving ChR2 expression with a viral injection in the cortex (Fig. 1, R and S).

In any case, future circuit mapping experiments accounting for several IT and PT subclasses will be required to dissect which PT subclasses are preferentially targeting ChIs and whether these interneurons and SPNs are contacted by the same or different presynaptic neuronal subpopulations. Understanding the intrastriatal connectivity of PT subclasses would, additionally, reveal whether during the second EPSC phase, acetylcholine enhances glutamate release from the same axons contacting ChIs or it acts onto other long-range afferents reaching the DLS. Given that nicotinic receptors containing the ligand binding α4 subunit are expressed in cortical, thalamic, and dopaminergic neurons (*68*–*71*) and that all of these neurons are able to release glutamate in the striatum, we must consider all of these afferents as potential sources of presynaptic release during the PT➔SPN EPSC second peak (Fig. 6, inset). However, because our experiments were centered in the DLS, where glutamate corelease from dopaminergic terminals has not been as established as in ventral striatal areas (*72*, *73*), the contribution of these axons to the EPSC second phase may be limited. In the future, exploring the selectivity of acetylcholine for gating individual populations of axons innervating the DLS will help in elucidating whether PT, IT, thalamic, dopaminergic, or other presynaptic inputs, alone or in specific combinations, underlie the second phase of excitation reported here. Distinguishing between these scenarios would have strong implications for addressing corticostriatal computations.

A drawback of our experimental approach is that, using the IT- and PT-ChR2-EYFP mice, we were not able to resolve the specific cortical area/areas innervating the recorded neurons. While it is widely accepted that, in the DLS, SPNs predominantly sample inputs form sensorimotor cortices, the cortical inputs to ChIs are less clear. Although long-range inputs to striatal ChIs have been classically proposed to be dominated by thalamic rather than cortical inputs (*43*, *64*, *74*, *75*), more recent studies proved a dense and heterogeneous corticostriatal connection to ChIs (*10*, *76*). Using retrograde viral tracing, those studies showed that, in addition to the previously described input from the cingulate cortex, the primary sensory, the primary motor and, in particular, the secondary motor cortex are also important sources of cortical input to ChIs in the dorsal striatum (*10*, *76*). Therefore, the specific contribution of each cortical area to the innervation of ChIs in the DLS, as well as their IT/PT differences, remains to be further explored. However, the innervation of the DLS is dominated by fibers from the sensorimotor areas, while axons from the cingulate cortex are scarce in this region (*11*, *77*). Thus, it is expected that, because of their dorsolateral location, both the SPNs and ChIs that we recorded were preferentially sampling motor and sensory cortical inputs.

SPNs and ChIs also sample long-range inputs from the thalamic parafascicular nucleus (PFN) (*10*, *53*, *65*, *76*). Thalamostriatal axons from these nuclei are also capable of amplifying intrastriatal glutamate release from long-range afferents through ChIs (*53*). In this circuit, axons from PFN➔ChI neurons drive the release of acetylcholine that, acting onto presynaptic α6β2 nicotinic receptors, enhances glutamate release from PFN➔SPN projections originated in a different population of thalamic neurons (*53*). This mechanism, which resembles the one that we described, is exacerbated in a parkinsonian mouse model, and its reduction strongly ameliorates motor deficits (*53*). Thus, intrastriatal amplification of excitation seems to be a widespread mechanism, involved in both physiological and pathological brain states. In the future, it will be interesting to explore the interplay between this α6β2-dependent thalamostriatal input amplification and the one that we described through α4-containing receptors, as well as the changes that the PT➔ChI➔glutAx➔SPN motif undergoes in pathological situations.

Besides amplifying excitation, the activation of the PT➔ChIs➔glutAx➔SPNs motif could gate a window for inducing long-lasting synaptic changes specifically at the activated Cx➔SPN contacts, by increasing local levels of acetylcholine. Changes in acetylcholine concentrations are believed to determine the occurrence and the sign (potentiation or depression) of long-term plasticity upon some pre- and postsynaptic activation combinations through muscarinic receptors (*78*–*80*) and by modulating dopamine levels (*43*, *81*, *82*). Dopamine has long been implicated as a key determinant of corticostriatal plasticity onto SPNs (*18*, *19*, *82*), and acetylcholine plays a crucial role in the striatum by modulating the local release of dopamine through nicotinic receptors, independently of distant somatic spiking (*43*). Thus, ChIs sit in a strategic position to orchestrate the events underlying corticostriatal plasticity onto SPNs (*62*, *78*). In this manner, the PT➔ChIs➔glutAx➔SPNs motif described here may provide a circuit mechanism supporting the delivery of a permissive signal for corticostriatal plasticity onto action-specific SPNs (*62*), conveyed by temporo-spatially restricted changes in striatal levels of acetylcholine, glutamate, and maybe dopamine when movement is executed. The activation of ChIs silences neighboring ChIs by feedback inhibition, further coordinating spatiotemporal acetylcholine fluctuations (*83*, *84*). Therefore, our work supports a model where PT long-range axons, besides directly selecting or invigorating the execution of a specific action by recruiting SPNs encoding for that action, could also gate plastic changes in corticostriatal synapses onto those SPNs by selectively activating specific ChIs.

In conclusion, our work dissects the IT and PT corticostriatal connectivity to ChIs, uncovering a circuit motif that biases ChI activation toward PT putative motor command signals. Together, our results propose a model where PT long-range axons, besides directly selecting or invigorating the execution of a specific action by recruiting SPNs encoding for that action, gate corticostriatal plasticity in specific synapses onto those SPNs. Therefore, the results presented here provide new insights into the polysynaptic impact of corticostriatal IT and PT signals onto SPNs, with potential implications for movement, plasticity, and learning. New research will be necessary to understand how other striatal microcircuit players sample and integrate long-range IT and PT inputs and their implications. Moreover, future studies assessing pathway-specific roles in vivo, where intact corticostriatal axons connected to their parental soma could behave differently, changing the dynamics that we observed in vitro, will help in tackling these complex corticostriatal circuits underlying motor learning and behavior.

## MATERIALS AND METHODS

### Animals

All procedures followed the Champalimaud Center for the Unknown Ethics committee guidelines, approved by the Portuguese Veterinary General Board (ref. no. 0421/000/000/2014). Both male and female transgenic mice ranging from 42 to 74 days of age were used. Mice were allocated to their experimental groups according to their genotype and age, so the age of the recorded neurons from IT and PT cohorts at each experimental condition were balanced (table S1). Animals were group-housed on a 12-hour light/dark cycle with ad libitum access to food and water. IT-cre [Tlx3 line, STOCK *Tg(Tlx3-cre)PL58Gsat/Mmucd*, RRID (Research Resource IDentifiers): MMRRC_036670-UCD, GENSAT (Gene Expression Nervous System ATlas), www.gensat.org/], PT-cre [OE25 line, STOCK Tg(Chrna2-cre)OE25Gsat/Mmucd, RRID: MMRRC_036502-UCD, GENSAT], and ChAT-Cre (*B6;129S6^Chattm2(cre)Lowl^/J*; Jackson Laboratory, #006410) were crossed with a cre-dependent ChR2-EYFP line [Ai32, B6;129S-Gt(ROSA)26Sor^tm32(CAG-COP4*H134R/EYFP)Hze^/J; Jackson Laboratory, #012569] generating double transgenic lines expressing ChR2-EYFP in specific neuronal populations. In some cases, triple transgenic mice were used by crossing IT- or PT-cre lines with Ai32 line and a transgenic BAC Drd1a-tdTomato line [*B6.Cg-Tg(Drd1a-tdTomato)6Calak/J*; Jackson Laboratory, #016204]. All lines were on C57BL/6 background by backcrossing with C57BL/6J inbred mice for at least eight generations.

The mouse strains used for this research project, STOCK *Tg(Tlx3-cre)PL58Gsat/Mmucd* (RRID: MMRRC_036670-UCD) and STOCK *Tg(Chrna2-cre)OE25Gsat/Mmucd* (RRID: MMRRC_036502-UCD), were obtained from the Mutant Mouse Resource and Research Center (MMRRC) at the University of California at Davis, a National Institutes of Health (NIH)–funded strain repository, and were donated to the MMRRC by N. Heintz, Rockefeller University, GENSAT, and C. Gerfen, NIH, National Institute of Mental Health.

### Slice preparation

Mice from 42 to 74 days of age were decapitated after deeply anesthetized with isoflurane. Brains were then dissected in ice-chilled choline chloride solution (110 mM choline chloride, 25 mM NaHCO_3_, 25 mM d-glucose, 11.6 mM sodium ascorbate, 7 mM MgCl_2_, 3.1 mM sodium pyruvate, 2.5 mM KCl, 1.25 mM NaH_2_PO_4_, and 0.5 mM CaCl_2_, bubbled with 95% O_2_/5% CO_2_), and coronal slices were cut (300-μm thickness) using a Leica VT1200S vibratome. Slices were incubated at 37°C in artificial cerebrospinal fluid (ACSF) (127 mM NaCl, 25 mM NaHCO_3_, 25 mM d-glucose, 2.5 mM KCl, 2 mM CaCl_2_, 1 mM MgCl_2_, and 1.25 mM NaH_2_PO_4_, bubbled with 95% O_2_/5% CO_2_) for 30 min before starting recording.

### Electrophysiology and photostimulation

Data were recorded using a Multiclamp 700B amplifier (Molecular Devices), digitized with a Digidata 1440 (Molecular Devices), and acquired at 10 kHz with pClamp 10 software (Molecular Devices). Neurons were recorded using borosilicate pipettes (resistance, 3 to 5 megohms; Harvard Apparatus) filled with internal solution containing the following: 135 mM potassium gluconate, 10 mM sodium phosphocreatine, 10 mM Hepes, 3 mM sodium l-ascorbate, 4 mM MgCl_2_, 4 mM Na_2_ATP, 0.4 mM Na_2_GTP, and 0.025 mM Alexa Fluor 594 (Molecular Probes); pH 7.2; 290 mOsm. In ChI-SPN experiments, biocytin (0.2%, Sigma-Aldrich) was added to the internal solution for neuronal reconstructions. All recordings were performed in normal ACSF preheated at 37°C and perfused at 1.5 to 2 ml/min rate. SPNs and ChIs were voltage-clamped close to their physiological membrane potential at −80 and − 55 mV, respectively. All recorded neurons were at least at 40-μm depth from the slice surface. For each photostimulation trial, input resistance was monitored with a hyperpolarizing test pulse. After each experiment, SPN identity was confirmed by imaging their morphology and dendritic spines (filled with Alexa Fluor 594; fig. S2A) at 60× with a BX61WI Olympus microscope, with a galvanometer-based scanning system (Bruker) and a two-photon Ti:sapphire laser (820 nm for imaging Alexa Fluor 594; Coherent), controlled by PrairieView software (Bruker). Somatic area and distance between ChIs and SPNs within the same neuronal pair were measured from these images using ImageJ/Fiji (NIH). ChI resting membrane potential was measured at break-in. Metrics from ChI spikes (interspike interval and half-width) and sag were extracted from current-clamp recordings by running a set of 15 square current steps (1-s duration, 20-pA increase, starting at −160 pA) before bath application of 4-AP and TTX. The position of the recorded neurons in the DLS was visually confirmed at 10×. All neurons were recorded from left DLS.

Wide-field photostimulation was performed using two fiber-coupled ~460-nm light-emitting diodes (LEDs; Doric Lenses) attached to the 60× microscope objective (~90° apart from each other, light incidence ~30° to the slice horizontal plane), to standardize the distance from the light source to the slice across experiments. To maximize the amount of light reaching the slice below the objective, the intensity of the illumination was fixed at maximum power (~10 and ~14 mW at the tip of the fiber for each LED) with an SLC-SA/SV/AA/AV series LED controller (Mightex, Canada). This photostimulation configuration required longer light pulses compared to those typically illuminating the slice directly through the objective. Thus, using PrairieView software, the light pulse duration was varied to elicit responses spanning the range of EPSC amplitudes explored. Full-field photostimulation through the objective lens in fig. S1 was performed using an ocular-mounted 470-nm LED (LXML-PB01-0030, Lumileds) controlled with an SLC-SA/SV/AA/AV series LED controller (Mightex, Canada) and focused on the back aperture of the 60× objective, resulting in an illumination spot of diameter ~800 μm and full width at half maximum of ~116 μm at the imaging focal plane. Interstimulus interval was 30 s, aiming at maximizing ChR2 recovery after illumination (*48*). This photostimulation protocol stabilized the EPSC second phase upon repetitive illumination (fig. S3), ruling out the possibility of confounding nicotinic receptor desensitization throughout the experiments.

For laser photostimulation of inputs to ChI-SPN pairs, light was delivered using a Point-Photoactivation module (Bruker) coupled to a one-photon 473-nm laser (Coherent). The grid was positioned so that the soma of the recorded neuron located in the center of a horizontal line placed at one-third of the total height (Fig. 5D). Each grid location (spacing, 55 μm) was typically photostimulated twice, following a non-neighboring pattern, maximizing the distance between consecutive laser pulses and resulting in an interval of >6 min between illuminations in the same location (interlocation interval, 30 s; intergrid repetition interval, 1 min). At the beginning of each grid experiment, laser intensity and duration were calibrated with PrairieView software so that the total input charge for IT➔ChI and PT➔ChI EPSC were comparable (Results and Fig. 5, E and G).

### Data analysis

Electrophysiology traces were analyzed using MATLAB (MathWorks). Total EPSC charge was calculated as the integral of the trace in a window of 65 ms starting with photostimulation. EPSC first-peak amplitude was computed as the minimum value in a window between the start of illumination and 35 ms. To minimize confounding EPSC second peak with first peak in cases where the second peak had higher absolute amplitude than the first peak, the interval from the photostimulation until the detected peak was scrutinized. When an earlier peak (with amplitude > 20% of the detected peak) followed by a valley (with depth > 2% of the detected peak) was present, the earlier value was counted as the EPSC first.

The decay of the EPSC first phase was subtracted from the EPSC second phase by subtracting a two-term exponential model from the recorded trace. To exclude the datapoints corresponding to the detection window of the EPSC second phase from the model fit, we used data from two discontinuous periods (50 ms in total). We defined an initial 10-ms period, starting where the EPSC first peak decayed 5% of its amplitude and finishing with the start of the detection window, and a second 40-ms period beginning immediately after that window. Thus, the second-phase detection period was restricted to a fixed-size window spanning from 10 to 32 ms after the EPSC first peak decayed to 95% of its amplitude. Within this window, EPSC second-phase peak amplitude and charge were computed as the minimum value and the integral of the subtracted trace, respectively. EPSC second phase was detected when the subtracted trace crossed a negative threshold (threefold the SD of baseline period) within the detection window. EPSC first-phase charge was calculated by subtracting the EPSC second-phase charge from the total EPSC charge. Charge ratio was calculated by dividing the charge of the EPSC second phase over the total EPSC charge. Peak ratio was computed by dividing the EPSC second-peak amplitude over the EPSC first-peak amplitude. In pharmacological experiments, baseline conditions were computed once the amplitude of the EPSC first peak stabilized after an initial ramping up period (fig. S3). In fig. S3A, peak amplitude was normalized to the amplitude of the first trial, while charge ratio and peak ratio were normalized to the first detected EPSC second phase.

The relationship between the photostimulation duration and the amplitude of the EPSC first peak was determined by calculating the Pearson’s correlation coefficient for every SPN photostimulated with more than one illumination duration. For each SPN, the slope of the correlation was calculated as *r*(SD (*y*) / SD (*x*)).

Latencies to EPSC first and second peaks were computed from the onset of the illumination pulse. EPSC trial-by-trial and neuron-based analyses used the same analysis criteria. Response probability was calculated by dividing the number of detected second phases by the total number of photostimulations in the bin (trial-by-trial; Fig. 1, E and I, and fig. S8) or SPNs (neuron-based; Fig. 1Q). Modulation index was calculated using the formula: (*X* in ACSF *− X* in drug) / (*X* in ACSF + *X* in drug), where *X* was charge ratio or peak ratio (fig. S5) and EPSC first- or second-phase charge or EPSC first- or second-phase peak amplitude (fig. S4).

For ChAT-ChR2 experiments, EPSC charge was calculated as the integral of the trace in a window of 65 ms starting with photostimulation. EPSC peak amplitude was computed as the minimum value in a window between the start of illumination and 50 ms.

For each neuron in 4-AP and TTX experiments in Fig. 5, EPSCs recorded in the same location were averaged, and charge was computed as the integral of the average trace in a window of 45 ms starting with the light pulse. In fig. S8, traces were analyzed with the same parameters that in Fig. 1 for comparison. ChI/SPN input ratio was calculated by dividing the sum of all EPSC charges in the ChI over the sum of all EPSC charges in its paired SPN. ChI sag difference was calculated as the difference between the mean membrane potential from 5 ms at the beginning and 5 ms at the end (800 ms apart) of a 1-s hyperpolarizing current step of −160 pA.

### Pharmacology

PTX (100 μM, Tocris), MLA (1 μM, Sigma-Aldrich), DNQX (10 μM, Tocris) + APV (50 μM; Tocris), atropine (10 μM, TCI), BTX (100 nM, Tocris), CONO (10 nM, Tocris), DHβE (1 μM, Tocris), and 4-AP (100 μM, Sigma-Aldrich) + TTX (1 μM, Abcam) were bath-applied. The effects of the drugs on the responses were tested after 5 min of drug recirculation in the recording chamber. From the 10 MLA experiments in Fig. 2, in 8 cases, MLA was added alone; in 2 cases, MLA was added together, with the nicotinic blocker mecamylamine (100 μM, Sigma-Aldrich). Monosynaptic connectivity was tested after, at least, 10 min of 4-AP and TTX recirculation.

### Confocal imaging

IT-, PT-, or ChAT-ChR2-EYFP mice at ~60 days of age were intracardiacally perfused with 4% paraformaldehyde. Brains were removed and coronally sliced (50-μm thickness). Slices were mounted using Mowiol and imaged with a Zeiss LSM 710 confocal microscope.

### Immunostaining and neuronal reconstruction

Slices with biocytin-filled ChI-SPN pairs were fixed in 4% paraformaldehyde for at least 2 hours at 4°C. Slices were then rinsed in phosphate-buffered saline (PBS) and incubated with goat anti-ChAT primary antibody (1:2500 to 1:5000, Chemicon/Millipore catalog no. AB144P) in PBS with 0.4% Triton X-100 and 2% normal horse serum (NHS; Gibco/Thermo Fisher Scientific catalog no. 16050-130) for two overnights at 4°C. After several PBS washes, slices were incubated with donkey anti-goat Alexa Fluor 405–conjugated secondary antibody (1:1000, Abcam catalog no. ab175664) and streptavidin conjugated with Alexa Fluor 594 (1:200, Life Technologies catalog no. 16892) in PBS with 0.4% Triton X-100 and 2% NHS for 2 hours at room temperature. Slices were mounted using Mowiol and imaged using a Zeiss LSM 710 confocal microscope. Slices from ChAT-ChR2-EYFP mice followed a similar protocol, but on the second day, they were incubated with donkey anti-goat Alexa Fluor 594–conjugated secondary antibody (1:1000, Molecular Probes catalog no. A11058), rabbit anti–green fluorescent protein conjugated with Alexa Fluor 488 (1:1000, Molecular Probes catalog no. A21311), and 4′,6-diamidino-2-phenylindole (1:1000, Sigma-Aldrich). Colocalization of streptavidin and ChAT or ChR2-EYFP and ChAT was assessed using stacks of individual confocal planes at 20× and maximal intensity projections. The proportion of ChR2-EYFP^+^ neurons coexpressing ChAT was calculated after manually counting single-/double-labeled striatal neurons unilaterally in two slices per brain from seven ChAT-ChR2-EYFP mice (~200 neurons per mouse). Dendritic morphology of the recorded neurons was reconstructed and measured from tiles of 25× images using neuTube (*85*) and Simple Neurite Tracer (*86*) plugin from ImageJ/Fiji (NIH). Reconstruction traces in Fig. 5 were exported using the HBP Neuron Morphology Viewer (*87*).

### Stereotaxic surgery

For viral injection in Fig. 1 (R and S), PT-cre mice of 25 days were anesthetized with isoflurane (1 to 3%, plus oxygen at 1 to 1.5 liters/min) and head fixed using a stereotaxic frame (David Kopf Instruments, Model 962LS) over a heating pad (ATC1000, World Precision Instruments) at 35° to 37°C. A small craniotomy was drilled over the left M1 following the coordinates (from bregma): 0.55 mm anterior/1.5 mm lateral. A pulled glass capillary (Drummond Scientific, USA) with a beveled tip of ~20-μm size was then lowered until reaching 750 μm of depth from the brain surface. Three-hundred nanoliters of AAV5-EF1a-DIO-hChR2(H134R)-EYFP-WPRE-pA (University of North Carolina Vector Core, USA) was then delivered with a Nanoject II Injector (Drummond Scientific, USA) at 4.6 nl/5 s rate. The capillary was removed 8 min after the last injection pulse. The skin was closed using Vetbond tissue adhesive (3M, USA). The mice were placed over a heating pad and fully recovered from anesthesia before returning to their homecage.

### Statistical analysis

All statistical analyses were done with MATLAB (MathWorks). Because normality was not assumed or rejected using Kolmogorov-Smirnov test, nonparametric two-tailed Wilcoxon rank sum test or two-tailed Wilcoxon signed-rank test was used across the manuscript for independent and paired comparisons, respectively. Statistical significance was defined as follows: ns, nonsignificant; *P < 0.05; **P < 0.01; ***P < 0.001; and ****P < 0.0001. Sample size were not predetermined, but our groups are in line with previous studies (*53*). Sample size, specific statistical test, exact *P* value and additional information are detailed in the figure legends or Results. Data are presented as means ± SEM if not indicated otherwise.

## Supplementary Materials

### Other Supplementary Material for this manuscript includes the following:

CSV Data files
