# Supplementary material for: Pyramidal tract neurons drive amplification of excitatory inputs to striatum through cholinergic interneurons
Source: Sci Adv. 2022 Feb 9;8(6):eabh4315. doi: 10.1126/sciadv.abh4315 (PMC8827762; doi:10.1126/sciadv.abh4315)
Supplement: Supplementary file 1 — Figs. S1 to S8 Table S1 [file sciadv.abh4315_sm.pdf]

Science Advances
AAAS

Supplementary Materials for

Pyramidal tract neurons drive amplification of excitatory inputs to striatum through cholinergic interneurons

Nicolás A. Morgenstern*, Ana Filipa Isidro, Inbal Israely, Rui M. Costa*

*Corresponding author. Email: nicolas.morgenstern@neuro.fchampalimaud.org (N.A.M.); rc3031@columbia.edu (R.M.C.)

Published 4 February 2022, *Sci. Adv.* **8**, eabh4315 (2022)
DOI: 10.1126/sciadv.abh4315

The PDF file includes:

Figs. S1 to S8
Table S1

Other Supplementary Material includes the following:

CSV Data files

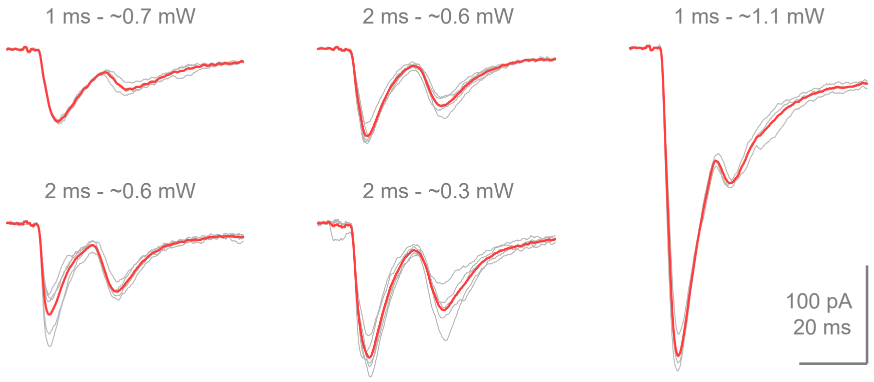

Fig. S1. Short duration light pulses reliably evoke biphasic PT→SPN EPSCs.

Example of EPSC traces from 5 different SPNs from 3 PT-ChR2-EYFP mice elicited by full-field photostimulation through the objective lens using light pulses of 1-2 ms and 0.3 to 1.1 mW. Thin light gray traces are the 3-5 individual trials corresponding to the thicker mean traces in red.

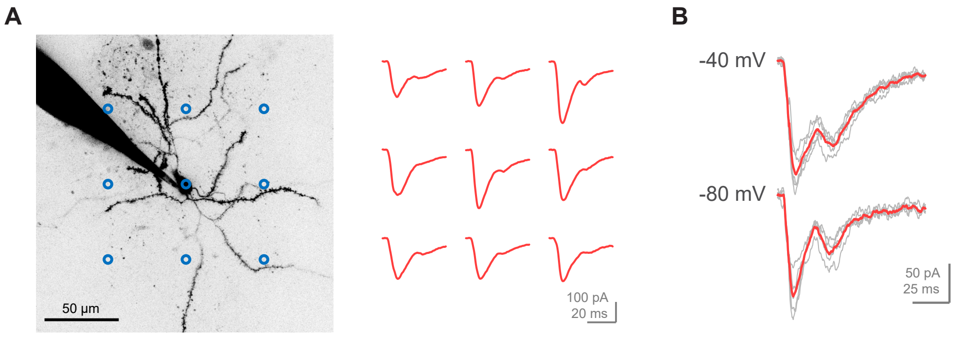

Fig. S2. PT→SPN EPSC 2nd peak is local and not mediated by GABA.

(**A**) Left, 2-photon image of a SPN showing the grid pattern used for focal photostimulation (blue circles). Right, individual EPSCs elicited when photostimulating PT axons at each grid location with a 473 nm 1-photon laser.

(**B**) Example of EPSCs elicited by the photostimulation of PT fibers while recording from a SPN in ACSF and held at membrane potentials below (-80 mV) and above (-40 mV) the calculated reversal potential for chloride (-75.6 mV). Thin light gray traces are the five individual trials corresponding to the thicker mean traces.

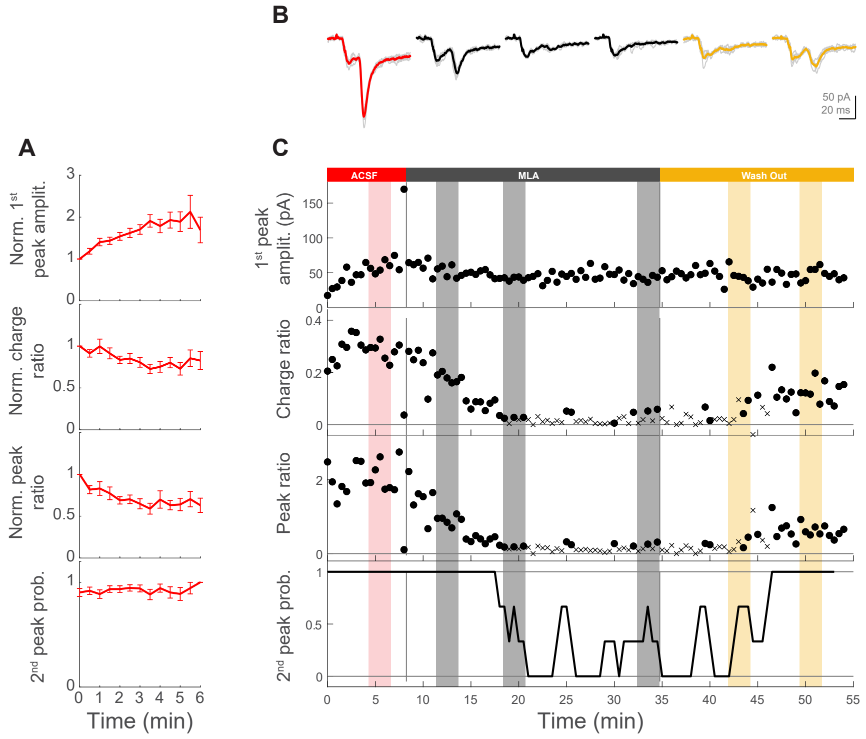

Fig. S3. Metrics extracted from PT→SPN EPSCs are reliable within and across experiments.

(**A**) From top to bottom: plots showing the normalized amplitude of the 1st phase of the EPSC, the normalized charge ratio, the normalized peak ratio and the response probability for a 2nd peak as a function of time, for all SPNs recorded while activating PT inputs in ACSF. Data is mean ± SEM. For each SPN, data was normalized to its first trial. Each time point is the average of 11-62 SPNs from 30 PT-ChR2-EYFP mice.

(**B**) Example of an individual experiment showing EPSCs from a SPN when identically photostimulating PT fibers in different bath conditions: ACSF (red), MLA (black), and wash out with ACSF (yellow). Thin light gray traces are the five individual trials underlying the thicker mean traces.

(**C**) From top to bottom: plots showing the amplitude of the 1st phase of the EPSCs, the charge ratio, the peak ratio and the response probability for a 2nd peak as a function of time, for the whole experiment in B. Data points are individual trials elicited every 30 s (for charge ratio and peak ratio, circles: EPSC with 2nd peak; crosses: EPSC without 2nd peak). Response probability was calculated within a moving window of 3 consecutive trials. Shadowed areas highlight the individual trials (thin light gray traces in B) averaged for each bath condition (red: ACSF; black: MLA; yellow: Wash out).

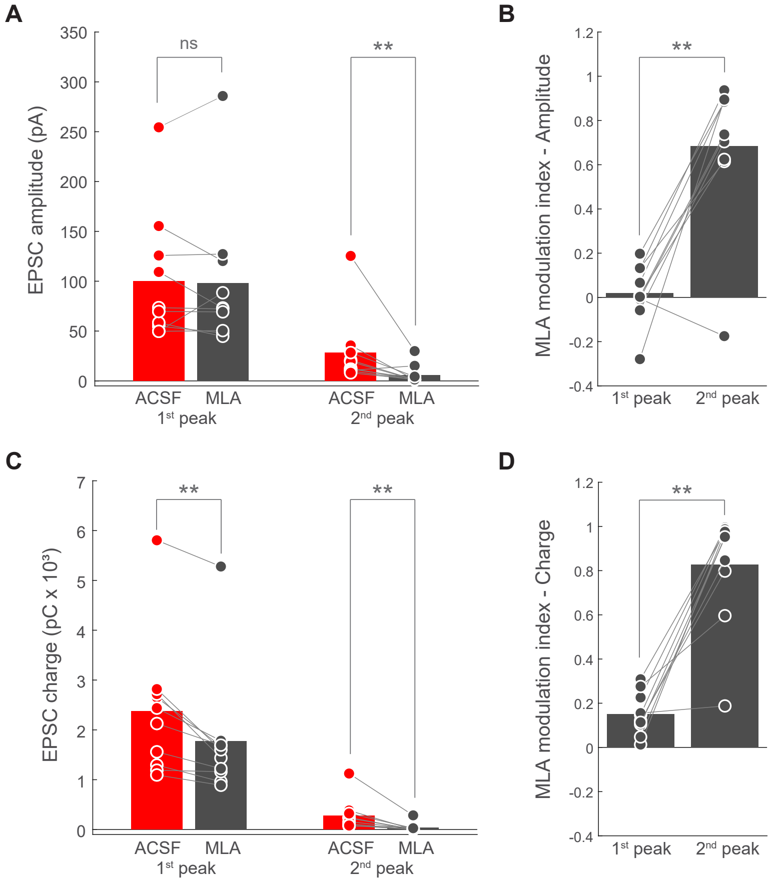

Fig. S4. PT→SPN EPSC 1st and 2nd peak are differentially modulated by nicotinic receptor blocker MLA.

(**A**) EPSC amplitude of 1st and 2nd peak for individual SPNs in ACSF (red) and MLA (black) conditions. n=10 SPNs from 7 PT-ChR2-EYFP mice. Bars represent mean. 1st peak p=0.69531; 2nd peak p=0.0039063; Wilcoxon signed-rank test.

(**B**) MLA modulation index of the amplitude of the 1st and 2nd peak for individual SPNs. n=10 SPNs from 7 PT-ChR2-EYFP mice. Bars represent mean. p=0.0039063; Wilcoxon signed-rank test.

(**C**) EPSC charge of 1st and 2nd peak for individual SPNs in ACSF (red) and MLA (black) conditions. n=10 SPNs from 7 PT-ChR2-EYFP mice. Bars represent mean. 1st peak p=0.0019531; 2nd peak p=0.0019531; Wilcoxon signed-rank test.

(**D**) MLA modulation index of the charge of the 1st and 2nd peak for individual SPNs. n=10 SPNs from 7 PT-ChR2-EYFP mice. Bars represent mean. p=0.0019531; Wilcoxon signed-rank test.

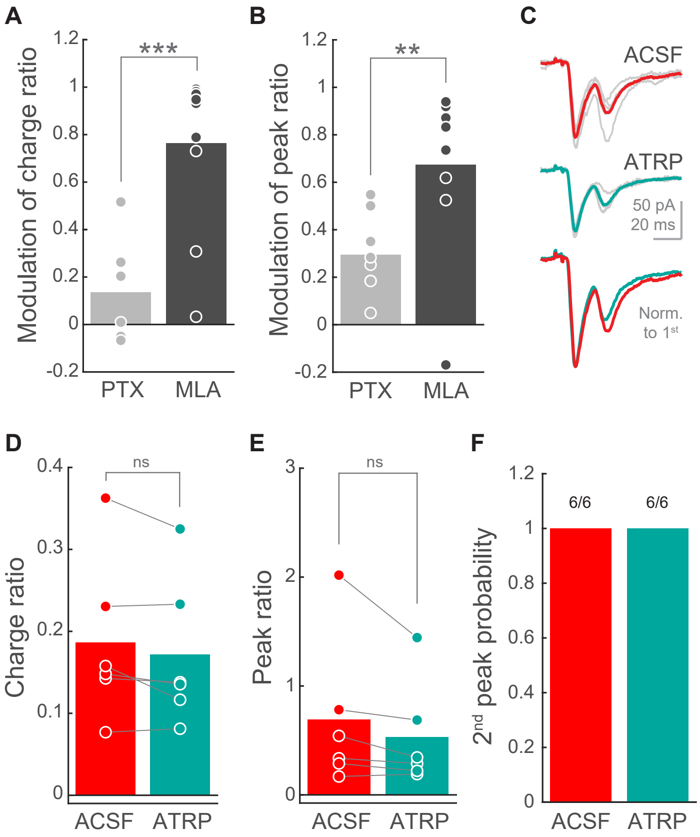

Fig. S5. PT→SPN EPSC second peak is not mediated by muscarinic receptors.

(**A, B**) Modulation index (**Methods**) of charge ratio (A) and peak ratio (B) for individual SPNs in PTX (gray) or MLA (black) conditions. PTX, n=8 SPNs from 5 PT-ChR2-EYFP mice; MLA, n=10 SPNs from 7 PT-ChR2-EYFP mice. Bars represent mean. p=0.00086841 (A); p=0.0043878 (B); Wilcoxon rank sum test.

(**C**) EPSCs from a representative SPN when photostimulating PT fibers in ACSF (top) or atropine (ATRP, middle). Thin light gray traces are the five individual trials corresponding to the thicker mean traces. Bottom, mean traces normalized to the first peak.

(**D**) Charge ratio for individual SPNs in ACSF and atropine. n=6 SPNs from 3 PT-ChR2-EYFP mice. Bars represent mean. p= 0.15625; Wilcoxon signed-rank test.

(**E**) Peak ratio for individual SPNs in ACSF and atropine. n=6 SPNs from 3 PT-ChR2-EYFP mice. Bars represent mean. p= 0.0625; Wilcoxon signed-rank test.

(**F**) 2nd peak response probability upon photostimulation of PT fibers for individual SPNs before and after the addition of atropine. n=6 SPNs from 3 PT-ChR2-EYFP mice.

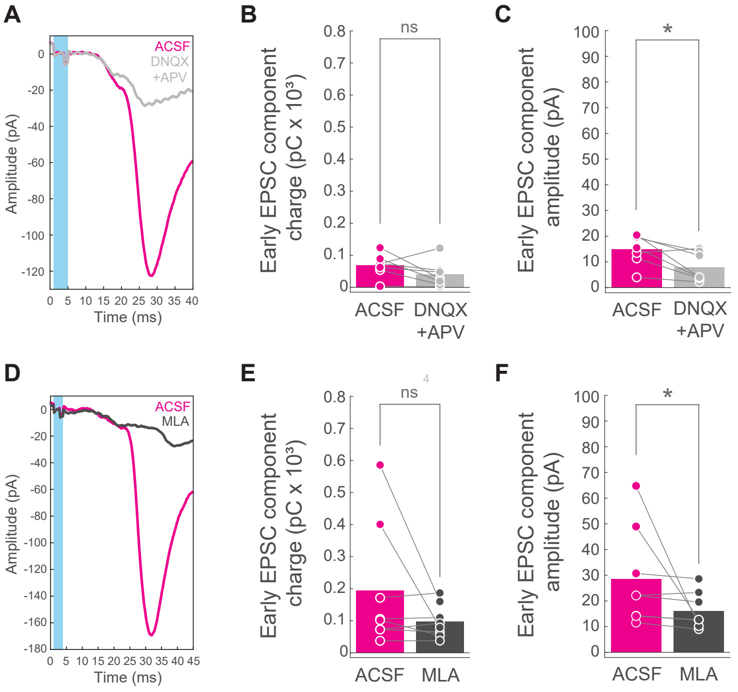

Fig. S6. ChI→SPN EPSCs peak is preceded by a small inward current component.

(**A**) Expanded view of the EPSCs in **Figure 3A**. Traces are the average of five individual trials. Blue shaded area represents the time (4 ms) the blue light was on.

(**B, C**) Early EPSC component charge (B) and amplitude (C) for individual SPNs in ACSF or DNQX and APV conditions. n=7 SPNs from 4 ChAT-ChR2-EYFP mice. Bars represent mean. p=0.15625 (B); p=0.03125 (C); Wilcoxon signed-rank test.

(**D**) Expanded view of the EPSCs in **Figure 3D**. Traces are the average of five individual trials. Blue shaded area represents the time (3 ms) the blue light was on.

(**E, F**) Early EPSC component charge (E) and amplitude (F) for individual SPNs in ACSF or MLA conditions. n=8 SPNs from 4 ChAT-ChR2-EYFP mice. Bars represent mean. p=0.46094 (E); p=0.015625 (F); Wilcoxon signed-rank test.

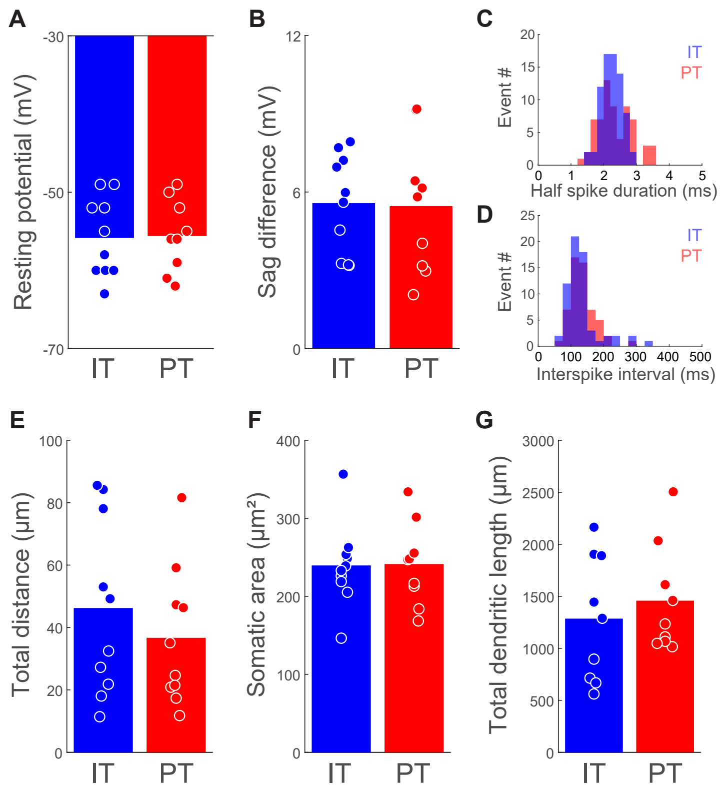

Fig. S7. ChIs recorded from IT- and PT-ChR2-EYFP mice belong to a morphofunctional homogenous population.

(**A**) Resting membrane potential of ChIs recorded from IT- or PT-ChR2-EYFP mice. Bars represent mean. IT: n=10 ChIs from 10 mice; PT: n=9 ChIs from 9 mice; p=0.9836; Wilcoxon rank sum test.

(**B**) Difference in membrane potential between the start and the end of the sag evoked upon a 1 s hyperpolarizing current step of -160 pA from ChIs recorded from IT- or PT-ChR2-EYFP mice. Bars represent mean. IT: n=10 ChIs from 10 mice; PT: n=9 ChIs from 9 mice; p=0.7197; Wilcoxon rank sum test.

(**C**) Histogram showing the distribution of half spike durations upon a 1 s depolarizing pulse of +60 pA from the ChIs recorded from IT- or PT-ChR2-EYFP mice. IT: n=74 spikes from 10 ChIs from 10 mice. PT: n=65 spikes from 9 ChIs from 9 mice; p=0.3982; Wilcoxon rank sum test, z=0.84.

(**D**) Histogram showing the distribution of inter-spike intervals upon a 1 s depolarizing pulse of +60 pA from the ChIs recorded from IT- or PT-ChR2-EYFP mice. IT: n=64 intervals from 10 ChIs from 10 mice. PT: n=56 intervals from 9 ChIs from 9 mice; p=0.18065; Wilcoxon rank sum test, z=1.34.

(**E**) Distance between an individual ChI and its paired SPN recorded from IT- or PT-ChR2-EYFP mice. Bars represent mean. IT: n=10 pairs from 10 mice; PT: n=10 pairs from 9 mice; p=0.42736; Wilcoxon rank sum test, z=0.79.

(**F**) Somatic area of ChIs from IT- or PT-ChR2-EYFP mice. Bars represent mean. IT: n=10 ChIs from 10; PT: n=9 ChIs from 9 mice; p=0.96824, Wilcoxon rank sum test.

(**G**) Total dendritic length of ChIs recorded from IT- or PT-ChR2-EYFP mice. Bars represent mean. IT: n=9 ChIs from 9 mice; PT: n=9 ChIs from 9 mice; p=0.48943; Wilcoxon rank sum test.

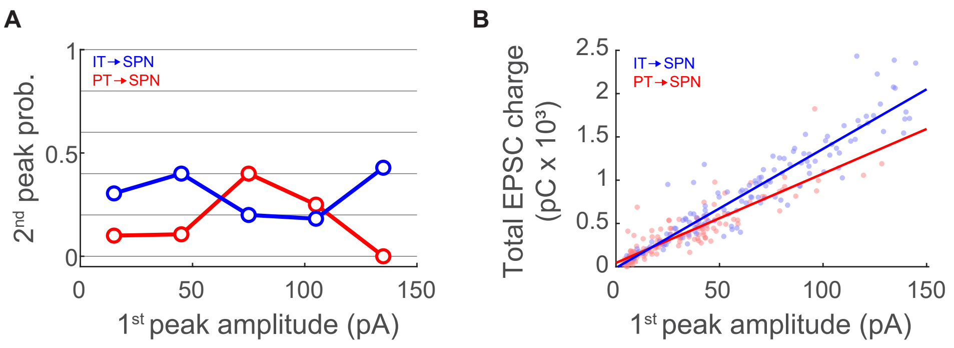

Fig. S8. 4-AP and TTX abolish the differences between IT- and PT→SPN EPSCs

(**A**) Probability of evoking a 2nd peak as a function of the 1st peak amplitude when photostimulating IT (blue) or PT (red) axons. Data is binned in 5 bins of 30 pA. The number of trials with 2nd peak/total number of trials for each bin is: IT, 7/23, 14/35, 7/35, 4/22, 6/14 from 10 SPNs from 10 IT-ChR2-EYFP mice; PT, 9/90, 5/47, 4/10, 1/4, 0/1 from 10 SPNs from 9 PT-ChR2-EYFP mice. See **Figure 1I** for comparison. Note that the biphasic events in these experiments are likely to be similarly overestimated for IT- and PT→SPN EPSCs due to the intrinsic occurrence of multiphasic events in these pharmacological conditions (49).

(**B**) Total charge of the EPSC as a function of the amplitude of its 1st peak. Each point represents an individual trial. n=129 trials from 10 SPNs from 10 IT-ChR2-EYFP mice (blue). n=152 trials from 10 SPNs from 9 PT-ChR2-EYFP mice (red). Solid lines are a linear fit for each group. Slope with 95% confidence interval (CI): IT, 13.75 pC/pA (12.79, 14.72); PT, 10.31 pC/pA (9.47, 11.15). Intersect with 95% CI: IT, -13.23 pC (-86.67, 60.21); PT, 44.82 pC (12.73, 76.9). See **Figure 1N** for comparison.

		IT age (days)	PT age (days)	Rank sum
Figure 1	Mean ± SEM	57.00 ± 1.75	59.15 ± 0.80	p = 0.8149
	Range	43 - 66	42 - 65	z = -0.2341
	n (SPNs)	26	65	
Figure 5	Mean ± SEM	65.10 ± 1.55	67.40 ± 2.18	p = 0.2879
	Range	58 - 72	53 - 74	z = -1.0627
	n (ChI-SPN pairs)	10	10	

Table S1. Neuronal age is similar between IT and PT groups within the same experimental condition.
